# Gut microbiota as a potential target of metabolic syndrome: the role of probiotics and prebiotics

**DOI:** 10.1186/s13578-017-0183-1

**Published:** 2017-10-25

**Authors:** Mingqian He, Bingyin Shi

**Affiliations:** Department of Endocrinology, The First Affiliated Hospital of Xi’an Jiaotong University, 277 West Yanta Road, Xi’an, 710061 Shaanxi People’s Republic of China

**Keywords:** Metabolic syndrome, Gut microbiota, Probiotics, Prebiotics, Potential therapeutic target

## Abstract

Metabolic syndrome (MS) comprises central obesity, increased plasma glucose levels, hyperlipidemia and hypertension, and its incidence is increasing due to changes in lifestyle and dietary structure in recent years. MS has been proven to be associated with an increased incidence of cardiovascular diseases and type 2 diabetes mellitus, leading to morbidity and mortality. In this manuscript, we review recent studies concerning the role of the gut microbiota in MS modulation. Manipulation of the gut microbiota through the administration of prebiotics or probiotics may assist in weight loss and reduce plasma glucose and serum lipid levels, decreasing the incidence of cardiovascular diseases and type 2 diabetes mellitus. To the best of our knowledge, short-chain fatty acids (SCFAs), bile salt hydrolase (BSH), metabolic endotoxemia and the endocannabinoid (eCB) system are essential in regulating the initiation and progression of MS through the normalization of adipogenesis and the regulation of insulin secretion, fat accumulation, energy homeostasis, and plasma cholesterol levels. Therefore, the gut microbiota may serve as a potential therapeutic target for MS. However, further studies are needed to enhance our understanding of manipulating the gut microbiota and the role of the gut microbiota in MS prevention and treatment.

## Background

Our lifestyle and dietary structure have significantly changed due to rapid economic development and improvements in quality of life, leading to the rapid occurrence of MS in recent years. MS is closely related to lifestyle and central obesity, serving as a risk factor for metabolic diseases, such as type 2 diabetes and cardiovascular disease. MS can be defined by the presence of abdominal obesity and any 2 of the following factors: increased fasting plasma glucose, increased TGs, reduced HDL cholesterol, and hypertension. In recent years, MS has spread across the globe. People with MS are twice as likely to die and three times as likely to have a heart attack or stroke than people without the syndrome. Thus, preventative and therapeutic strategies to reduce the morbidity and mortality caused by metabolic diseases are significant.

To date, the human intestinal microbiota has gained increasing interest for its equivocal impact on human health, such as its comprehensive physiological and pathological functions [1–4]. A plethora of microorganisms have colonized the gastrointestinal (GI) tract by the time that we are born, and they play a crucial role in building our future physiology and immunity, leading to homeostasis of the internal environment. The role of bacteria in shaping immunity and gut structure has emerged over the last decades. The human intestinal microbiota composition is the result of a bi-directional interaction between the host and its microbial consortium. Immune factors, such as secretory IgA and endogenous secretions, end up in the intestine and have been proven to affect the composition of the intestinal microbiota [5, 6]. In addition to these endogenous modulations, the composition and stability of the intestinal microbiota are determined by nutrition or other factors, such as probiotics, prebiotics, antibiotics, drugs, and diseases. Current studies suggest that manipulation of the gut microbiota could be a promising approach for the prevention and management of metabolic syndrome [7].

Probiotics (mainly bifidobacteria and lactobacilli) reside in the human colon, where they exert actions such as modulating colon micro-flora and immunogenic responses and producing certain materials; altogether, these functions improve the host’s health. Probiotics may help prevent infections, reduce cholesterol levels, promote vitamin and cytokine synthesis and inhibit cancer progression. The safety and efficacy of a given strain in the context of these properties must be scientifically demonstrated for it to be considered a probiotic. Prebiotics are described by Gibson and Roberfroid as non-digestible poli- or oligosaccharides (OS) that beneficially affect the host by selectively stimulating the growth and/or activity of one or a limited number of beneficial bacteria in the colon [8]. In combination, prebiotics and probiotic bacteria create synbiotics, which can provide even more benefits than probiotics or prebiotics alone. Here, we have reviewed recent studies concerning the role of the gut microbiota in metabolic syndrome and the effects of probiotic bacterial strains and prebiotics on the prevention or treatment of metabolic syndrome, such as their anti-obesity and anti-inflammatory effects and their ability to improve glycemic control and modulate serum lipids.

## Gut microbiome and metabolic syndrome

A wide variety of commensal microbes colonize our body surfaces and gut lumen. For example, there are more than 100 trillion commensal microbes classified into at least 1000 different species in our gastrointestinal tract. Nevertheless, our understanding of the diversity of the gut microbiota was largely limited and biased, and it showed that the gut microbiota is mainly composed of four phyla, namely, Firmicutes, Bacteroidetes, Actinobacteria, and Proteobacteria. The number of microbes in the gut microbiota is approximately ten times that of somatic cells in our body, and these microbes participate in most metabolic activities in vivo with noticeable effects. Moreover, the gut microbiota contains 600,000 genes [9], which is approximately 25 times more than the number of genes in our own genome, highlighting the existence of a highly complex microbiota ecosystem with the potential for profound effects on metabolism and immune function. Intestinal immune, nerve, and endocrine cells are tightly interlinked and form a highly complex gut ecosystem, along with the gut microbiota, through host-microbial crosstalk, which contributes to homeostatic balance in the host. Therefore, it was vital to understand the gut ecosystem by comprehensively analyzing the host, the gut microbiota, and their interactions.

The gut microbiota was thought to possess a variety of functions in human physiology and pathology. For example, it aids in host nutrition and energy harvest, vitamin production, and the fermentation of food components that are otherwise indigestible by the host [10–13]. It also contributes to intestinal epithelial homeostasis, the development of the immune system, drug metabolism, and protection against pathogens [14–17]. The gut microbiota can perform defensive functions in healthy individuals directly by impeding colonization by pathogens that are competing for space and nutrients or indirectly by producing antimicrobial compounds, volatile fatty acids and chemically modified bile acids. In this way, the indigenous gut bacteria are able to modify adverse conditions for the inoculation and development of enteric pathogens through the barrier effect or colonization resistance. They also exert a significant role in the pathogenesis of metabolic syndrome, as confirmed by studies conducted on humans and in animal models. An impairment of the fine balance between gut microbes and the host’s immune system could culminate in the intestinal translocation of bacterial fragments and the development of “metabolic endotoxemia” (caused by bacteria and/or bacterial fragments, such as lipopolysaccharides, which pass through the gut barrier into the blood), leading to systemic inflammation. Because these molecules can stimulate macrophage infiltration and activate the synthesis of inflammatory cytokines, an increase in cytokine signaling can inhibit protein synthesis and enhance catabolism. Current views suggest that low-grade chronic systemic inflammation contributes to the development of insulin resistance, diabetes, and obesity. The relative proportion of some major phyla of gut bacteria, such as Bacteroidetes and Firmicutes (a lower proportion of Bacteroidetes and higher abundance Firmicutes), was associated with metabolic syndrome [18]. Ferrer et al. conducted an investigation of gut microbial communities in fecal samples taken from an obese adolescent and a lean adolescent by analyzing the diversity of 16S rDNA amplicons, 22 Mbp of consensus metagenome sequences and the expression profiles of 613 distinct proteins. They found that in the obese gut, the phylum Firmicutes (94.6%) was more abundant in the total microbiota than Bacteroidetes (3.2%), whereas the lean gut showed a remarkable shift towards Bacteroidetes (18.9% of total 16S rDNA), which became the most active fraction (81% of proteins) [18]. These facts generally implicated the role of the gut microbiota in the pathophysiology of metabolic syndrome.

Diet-induced weight loss and bariatric surgery promoted significant changes in gut microbial composition and thus changed treatment strategies. Manipulation of the gut microbiota through the administration of prebiotics or probiotics can reduce intestinal low-grade inflammation and improve gut barrier integrity, thus ameliorating metabolic balance and promoting weight loss. Therefore, a comprehensive understanding of the gut microbiota manipulation and an assessment of risk factors in related disorders was necessary to generate therapeutic approaches to cure these diseases.

## History of probiotics and prebiotics

One approach to modulating the gut microbiota was the ingestion or administration of probiotics. The term “probiotic” originated from the Greek word meaning “for life” [19]. Probiotics are defined as living microorganisms that can exert beneficial health effects on the host by improving their intestinal microbial balance after entering the gut. The definition originally came from Elie Metchnikoff at the beginning of the 20th century. Elie Metchnikoff, the father of the probiotics concept, first referred to the properties of fermented milk (containing lactic acid bacteria) used by native, long-living Bulgarian populations and linked it to increased well-being. In his book, ‘The Prolongation of Life’, he illustrated the effects of responsible microorganisms, and his work laid the foundation for further studies on the positive effects of bacteria. The discovery of bifidobacteria in the microbiota of human milk-fed infants by Henri Tissier at the Pasteur Institute led to the recommendation to administer bifidobacteria to infants with diarrhea in the 1950s [20]. To date, the discovery and development of new probiotics still greatly relies on experiments, regardless of the fact that humans have been using probiotics for a long time. Bifidobacteria and/or lactobacilli, as well as other lactic acid bacteria, such as lactococci and streptococci, can be found in most of today’s probiotic products. Other promising probiotic strains include organisms of the bacterial genera Bacillus, Enterococcus, Escherichia, and Propionibacterium and of the yeast genus Saccharomyces. Generally, probiotics are considered essentially innocuous for human ingestion with limited reported cases of adverse events.

In addition to probiotics, the gut microbiota can be modulated via the administration of prebiotics. Gibson and Roberfroid put forward the prebiotic concept in 1995 [8]. Thereafter, the employment of specific non-digestible carbohydrates (NDO) devoted to modulation of the gut microbiota raised researchers’ attention. In 2008, the most recent definition of a prebiotic was formulated as a selectively fermented dietary ingredient that resulted in specific changes in the composition and activity of the gastrointestinal microbiota, thus conferring benefits on host health. Most scientific data were obtained using food ingredients/supplements belonging to two chemical groups, namely, inulin-type fructans (ITF) and galacto-oligosaccharides (GOS). These data repeatedly demonstrated selective stimulation of bifidobacteria growth, and in some cases, lactobacilli lead to a significant change in gut microbiota composition. The prebiotic concept and its health effects were extensively reviewed by Gibson and Roberfroid in 2010 [21]. To date, the most studied prebiotics are the fructooligosaccharides (FOS) inulin and oligofructose [22, 23]. Nevertheless, many other OS, such as xylo-oligosaccharides (XOS), pectic oligosaccharides (POS), cyclodextrins, palatinose and OS from pullulan, are also important prebiotic candidates.

Because of these health effects stemming from the alteration of intestinal microbial balance, probiotics and prebiotics began to blossom in the late 1800s and early 1900s. There is a long history of human consumption of probiotics (particularly lactic acid bacteria and bifidobacteria) and prebiotics as natural components of food or as fermented foods. This longstanding use highlights a growing recognition of the role of probiotics and prebiotics in modulating the metabolic activities of the human gut microbiota and regulating the immune system, thus improving the host’s health. Probiotic manipulation of the microbiota may therefore be complementary to the application of prebiotic supplementation. The combination of both pre- and probiotics, also referred to as synbiotics, constitutes another nutritional tool for modulating the microbiota. Ishizuka et al. demonstrated a synergistic effect of synbiotics in combination with Bifidobacterium breve and raffinose on intestinal epithelial proliferation in rats.

## Probiotic and prebiotic consumption can ameliorate MS components

### The anti-obesity effect

Most studies regarding the “anti-obesity” effect of probiotics performed in rodents were achieved with members of the genus Lactobacillus. Lactobacillus strain administration led to several metabolic benefits in rodents: a reduction in adipocyte cell size and body fat in high-fat diet fed mice [24], a reduction in fat mass, and restriction of excessive body weight gain [25]. Diet-induced obese mice and diet-induced overweight rats showed a reduction in body weight gain after they were fed specific Lactobacilli [26, 27]. It was reported that the administration of *Lactobacillus gasseri* BNR17 could reduce body weight and fat mass gain in high-sucrose diet-induced obese rodents and fasting glycemia in db/db mice. Other studies showed that *L. gasseri* SBT2055 (LG2055) could decrease fat mass and adipocyte size in rodents [28–30]. For example, Miyoshi et al. revealed that LG2055 administration resulted in a significant reduction in body weight and fat tissue mass (epididymal and perirenal/retroperitoneal) and inhibited the up-regulation of pro-inflammatory gene expression in adipose tissue, which might be a possible mechanism underlying the anti-obesity effect of LG2055 [29]. Other in vivo studies showed that *L. rhamnosus* GG or *L. sakei* NR28 administration could decrease body weight gain and adipose tissue weight in mice. Both strains could down-regulate lipogenic gene expression in the liver [31]. These results suggested that in addition to effects on body weight and fat mass, the administration of probiotics could counteract some metabolic diseases related to obesity.

In addition to studies utilizing Lactobacillus species, several studies used specific Bifidobacterium strains alone, such as *Bifidobacterium longum*, *B. adolescentis* and a combination of Bifidobacterium species (*B. pseudocatenulatum* SPM1204, *B. longum* SPM1205, and *B. longum* SPM1207). These studies showed that *Bifidobacterium* spp. could decrease body weight gain and adipose tissue in high-fat diet (HFD)-induced obese rats [32–34]. A recent study also demonstrated that administration of the strain *B. pseudocatenulatum* CECT7765 could ameliorate metabolic and immunologic obesity-associated alterations by reducing liver steatosis and the number of larger adipocytes and fat micelles in the enterocytes of obese mice [35].

The “anti-obesity” effects of probiotic use can interfere with intestinal functions. An example was the administration of engineered NAPE-expressing *Escherichia coli* Nissle 1917 bacteria for 8 weeks. Chen et al. demonstrated that incorporation of these modified bacteria in the drinking water of mice fed a high-fat diet resulted in dramatically lower food intake, adiposity, insulin resistance, and hepatosteatosis, whereas weight gain was inhibited in a polygenic mouse model of obesity (TallyHo mice) [36].

Prebiotic supplementation of obese animals (ob/ob mice, diet-induced obesity, obese Zucker or JCR:LA-cp rats) also decreased body weight gain, adipocyte size, adiposity, and insulin resistance [37, 38]. A high-fat diet-induced an accumulation of large adipocytes, promoted peroxisome proliferator activated receptor gamma (PPARγ)-activated differentiation factors and led to a huge increase in G-protein-coupled receptor 43 expression in subcutaneous adipose tissue. In HFD-fed mice, dietary supplementation with non-digestible/fermentable carbohydrates, such as ITF or arabinoxylans, could lessen adiposity [39]. Prebiotic treatment could lower adiposity by changing the gene expression pattern in white adipose tissue of obese mice (by acting on PPARγ and GPR43), leading to increased lipolysis, decreased adipogenesis, and an increased metabolic response to hormones such as leptin [37, 40]. In obese animals fed ITF (10% in the diet), a decrease in food intake and an increase in anorexigenic peptides [peptide YY (PYY) and glucagon-like peptide-1 (GLP-1)] through modulation of the production of gastrointestinal peptides could be detected. The increase suggested that the improvement of obesity and related diseases by fermentable carbohydrates could be mediated through modulation of the endocrine function of the gut. Recently, Dewulf et al. reported that ITF supplementation in high-fat diet fed male C57BL/6J mice increased fermentation in the cecum, which paradoxically counteracted HF diet-induced GPR43 overexpression in adipose tissue; this phenomenon correlated with a beneficial effect on adiposity and a potential decrease in PPARγ-activated processes [37].

Only a small number of studies focusing on human interventions were designed to analyze the effect of probiotic administration on body fat and weight [41, 42]. In a multicenter, double-blind, randomized, placebo-controlled clinical intervention trial, 87 subjects with a higher body mass index (BMI) (24.2–30.7 kg/m^2^) and abdominal visceral fat area (81.2–178.5 cm^2^) were randomly assigned to receive either fermented milk (FM) containing LG2055 (active FM; n = 43) or FM without LG2055 (control FM; n = 44); then, they were asked to consume 200 g/day of FM for 12 weeks. In the active FM group, abdominal visceral and subcutaneous fat areas significantly (P < 0.01) decreased from baseline by an average of 4.6 and 3.3%, respectively. Of these parameters, the reduction in visceral fat stood out because an excess accumulation of visceral fat was primarily involved in metabolic disorders, and visceral fat was more strongly correlated with most metabolic risk factors than subcutaneous fat. Body weight and other measures also decreased significantly (P < 0.001) as follows: body weight, 1.4%; BMI, 1.5%; waist, 1.8%; and hip, 1.5%. None of these parameters significantly decreased in the control group. The outcome of this study indicated that the probiotic LG2055 lowered abdominal adiposity, body weight and other measures, suggesting its beneficial influence on metabolic disorders [42].

It was proven that prebiotics contributed to weight loss and improved metabolic parameters, such as insulin resistance, in overweight or obese individuals [43]. Moreover, satiety, reduced energy and food intake, and increased levels of satiety peptides also resulted from the consumption of prebiotics in healthy human subjects [44]. For example, ingestion of ITF (8 g/day) for 1 year showed significant reductions in BMI and fat mass in non-obese young adolescents [45].

In clinical experiments, beneficial effects of prebiotic administration were observed, such as a reduction in BMI, waist circumference, fat mass, and insulin resistance [21, 45, 46]. The daily intake of yacon syrup in obese pre-menopausal women, which delivered 0.14 g of fructooligosaccharides per kg per day over 120 days, increased satiety sensation and defecation frequency and decreased body weight, waist circumference and body mass index [46]. In a subsequent clinical trial, short-chain inulin-type fructans given as a supplement for 12 weeks (21 g/day) decreased food intake, body weight gain and fat mass development, and an increase of plasma PYY levels and a drop in ghrelin after a meal were detected in otherwise healthy adults with a body mass index > 25 kg/m^2^, providing evidence that oligofructose supplementation has the potential to promote weight maintenance [43].

As previously known, the gut microbiota regulated obesity-related biological systems, such as nutrient supply, fat accumulation and energy storage [47, 48]. In addition, gut ecology could be influenced by insulin-type fructans, which also activated immune cells. Accumulating studies have indicated that insulin-type fructans decreased fat accumulation and body weight in vivo, such as in obese individuals [49–51].

### The effect on improving glycemic control

Oral administration of probiotics and/or prebiotics could decrease serum glucose levels. Specific animal models using diet-induced obese mice or diabetic mice have been commonly applied to evaluate the effects of probiotics on the characteristics of type 2 diabetes mellitus (T2DM) to report the beneficial effects of various strains of Lactobacilli [52]. In high-fructose fed rats, the anti-diabetic effect of probiotics was measured by feeding them probiotics containing *Lactobacillus acidophilus* and *Lactobacillus casei* [53]. Recently, Naito et al. described both the anti-diabetic and anti-inflammatory effects of *L. casei* in diet-induced obese mice. Yadav et al. also demonstrated that administration of dahi (yogurt on the Indian subcontinent) containing probiotic *L. acidophilus* and *L. casei* in male diabetic rats (induced by feeding 21% fructose in water) for 8 weeks significantly delayed the onset of glucose intolerance, hyperglycemia, hyperinsulinemia, and dyslipidemia and decreased oxidative stress [53].

*Akkermansia muciniphila* was of interest among the bacteria that could potentially be used for the amelioration of type 2 diabetes. The direct beneficial effects of this bacterium on glucose metabolism were identified in a diet-induced type 2 diabetes mouse model using *A. muciniphila* MucT (ATTC BAA-835) [54]. First, *A. muciniphila* decreased glucose-6-phosphatase (G6pc) mRNA expression to counteract fasting hyperglycemia in the mouse model [54]. This implied that *A. muciniphila* decreased gluconeogenesis in a diabetic mouse model. Furthermore, administration of live *A. muciniphila* could also alleviate glucose intolerance [54, 55]. However, additional studies were needed to clarify whether *A. muciniphila* could be used as a probiotic for type 2 diabetes patients or not.

The beneficial effects of the consumption of multispecies probiotic supplements on insulin resistance and metabolic profiles, including high-sensitivity C-reactive protein (hs-CRP), were also reported in diabetic patients. Asemi et al. utilized an oral supplement comprising seven viable and freeze-dried strains: *L. acidophilus*, *L. casei*, *L. rhamnosus*, *L. bulgaricus*, *Bifidobacterium breve*, *B. longum*, *Streptococcus thermophilus*, and 100 mg of fructooligosaccharide. Fifty-four diabetic patients aged 35–70 years were randomly assigned to take either a multispecies probiotic supplement (n = 27) or a placebo (n = 27) for 8 weeks. Between-group comparisons of fasting plasma glucose (FPG) revealed that consumption of probiotic supplements prevented a rise in FPG (+ 28.8 ± 8.5 for placebo vs. + 1.6 ± 6 mg/dl for probiotic group, P = 0.01). Mean changes in serum hs-CRP were significantly different between the two groups (− 777.57 for the probiotic group vs. + 878.72 ng/ml for the placebo group, P = 0.02). Probiotic supplementation led to a significant increase in plasma GSH levels compared to those with the placebo (240.63 vs. − 33.46 µmol/l, P = 0.03). The results of this study indicated that multispecies probiotic supplementation for 8 weeks in diabetic patients prevented a rise in FPG and resulted in a decrease in serum hs-CRP and an increase in plasma total glutathione, r-glutamyl cysteinyl + glycine (GSH) compared with placebo [56].

Probiotic yogurt supplementation controlled glycemic level (reduced fasting blood glucose and glycated hemoglobin) in type 2 diabetic patients. After consuming probiotic yogurt (*L. acidophilus* La5 and Bifidobacterium lactis Bb12) for 6 weeks at the dose of 300 g/day, T2D patients experienced a decrease in fasting blood glucose and HbA1. Additionally, probiotics could promote antioxidation in T2DM patients. An increase in erythrocyte superoxide dismutase, glutathione peroxidase activities, and total antioxidants could be detected in the group supplemented with probiotic yogurt [57].

Sasaki et al. showed that type 2 diabetic patients treated with transglucosidase (which generates prebiotic fibers, including oligosaccharides, from dietary starch in the human GI tract) experienced reduced levels of hyperglycemia and body weight gain. These effects were mediated by increased gut production of oligosaccharides and alteration of the gut microbiota composition (increased Bacteroidetes-to-Firmicutes ratio) [58].

### The effect on modulating serum lipids

Ann and Spoerry observed the hypocholesterolemic activity of fermented milk in a Maasai tribe located in Kenya. Animal and human models have since been used to evaluate the effects of probiotic microorganisms on serum lipid levels, and probiotic benefits have been emphasized over the last 40 years. Accumulating studies have shown that well-established probiotics, prebiotics and synbiotics possess hypocholesterolemic effects and other effects that modulate serum lipids in humans and animals.

It was reported that probiotic administration could modulate lipid metabolism in animal models, such as in diet-induced obese mice, hypocholesterolemic mice, and hypercholesterolemic rats. Kumar et al. suggested that the indigenous *Lactobacillus plantarum* Lp91 strain had the potential to be explored as a probiotic in the management of hypercholesterolemia by reporting the hypocholesterolemic effect of *L.* *plantarum* in rats fed a hypocholesterolemic diet [59]. In addition, Mohania et al. observed that the supplementation of probiotic dahi prepared by *L. plantarum* Lp9 might have the therapeutic potential to decrease plasma, hepatic, and aortic lipid profiles and attenuate diet-induced hypercholesterolemia in rats fed a hypercholesterolemic basal diet [60]. Nguyen et al. demonstrated that total serum cholesterol and triglycerides were significantly reduced (by 7 and 10%, respectively) in hypocholesterolemic mice that ingested *L. plantarum* PH04 for 14 days [61]. The administration of probiotic strain *Lactobacillus* *curvatus* HY7601(CU), combined or not combined with *L. plantar*um KY1032(PL), reduced plasma cholesterol levels and hepatic lipid content (TGs and cholesterol) in mice fed a high-fat high-cholesterol diet (HFCD) [62].

Another focus of the research community was the role of prebiotics in the prevention of cardiovascular disease (CVD) in animal models. Studies by Rault-Nania et al. on apo E-deficient mice demonstrated that the addition of long-chain inulin in the diet of mice inhibited the formation of atherosclerotic plaques; this effect is probably related to changes in lipid metabolism. Both long-chain inulin and an oligofructose-enriched inulin significantly lowered hepatic cholesterol concentrations compared with the control diet (P < 0.05) [63]. The addition of inulin in the diet of rats induced higher excretions of fecal lipids and cholesterol compared to the excretions of rats in the control group. This increased level of excretion was attributed primarily to reduced cholesterol [64]. The administration of a synbiotic food containing *L. acidophilus* ATCC 4962, fructooligosaccharide, inulin and mannitol in hypercholesterolemic pigs for 8 weeks resulted in reductions in serum triglycerides and total- and low density lipoprotein (LDL)-cholesterol levels as well as an increased HDL-cholesterol concentration [65].

Ataie-Jafari et al. evaluated a group of people with mild to moderate hypercholesterolemia and reported that after consumption of probiotic yogurt (fermented with a starter composed of *L. acidophilus* and *Bifidobacterium lactis* in addition to the bacteria in ordinary yogurt) for 6 weeks, blood cholesterol rates were significantly lowered, whereas other blood lipid indices did not show any significant differences compared with those of the group that consumed traditional yogurt [66]. Similarly, Jones et al. demonstrated that the consumption of a yogurt containing microencapsulated bile salt hydrolase-active *Lactobacillus reuteri* NCIMB 30242, which was taken twice per day during a 6-week period, was effective at reducing LDL-cholesterol, total cholesterol and non-HDL cholesterol in hypercholesterolemia adults; this treatment appeared to be superior to traditional probiotic therapy [67]. Anderson et al. utilized a probiotic called *L. acidophilus* L1 and showed that daily consumption of 200 g of fermented milk (FM) containing *L. acidophilus* L1 for 3 weeks was accompanied by a 2.4% (P < 0.05) reduction of serum cholesterol concentration compared to that of the placebo group [68]. Fukushima et al. indicated that a mixture of organisms (a probiotic mixture) comprised of Bacillus, Lactobacillus, Streptococcus, Clostridium, Saccharomyces, and Candida effectively reduced total cholesterol and liver cholesterol compared to individual bacteria strains. The supplied mixed-bacteria and *L. acidophilus* groups exhibited a 23–57% decrease in cholesterol concentrations in the liver. Additionally, the serum total cholesterol in the supplied mixed-bacteria group was reduced by 15–33% compared with that in the single-bacteria supplemented groups [69].

Consumption of prebiotics was shown to improve lipid metabolism in healthy volunteers. Brighenti et al. showed that inulin seemed to have a lipid lowering potential in normolipidemic men. When normolipidemic individuals consumed cereal containing 18% inulin on a daily basis without any other dietary restrictions, total plasma cholesterol and triacylglycerols decreased by 7.9 ± 5.4% (P < 0.05) and 21.2 ± 7.8% (P < 0.005), respectively [70]. Recently, Russo et al. concluded that an intake of 11% inulin-enriched pasta in healthy young male volunteers for 5 weeks improved HDL cholesterol and the total cholesterol/HDL-cholesterol ratio [71].

A significant reduction in serum total- and LDL-cholesterol levels was also seen with intake of a synbiotic containing *L. gasseri* and inulin among hypercholesterolemic patients after 12 weeks. Schaafsma et al. found that consumption of milk (fermented by yogurt starters and *L. acidophilus* and containing 2.5% fructooligosaccharides) by adult male volunteers for 3 weeks significantly lowered values of serum total cholesterol (P < 0.001), LDL-cholesterol (P < 0.005), and the LDL/HDL-ratio (P < 0.05) by 4.4, 5.4 and 5.3%, respectively [72].

Some researchers have investigated the effects of prebiotics on cholesterol levels, but the results have not been consistent. Balcázar-Muñoz et al. reported that the oral consumption of inulin (7 g/day) for 4 weeks by dyslipidemic obese subjects led to a significant reduction of total cholesterol (248.7 ± 30.5 and 194.3 ± 39.8 mg/dl; P = 0.028), LDL, cholesterol (136.0 ± 27.8 and 113.0 ± 36.2 mg/dl; P = 0.028), very low density lipoproteins (VLDL) (45.9 ± 18.5 and 31.6 ± 7.2 mg/dl; P = 0.046) and triglyceride concentrations (235.5 ± 85.9 and 171.1 ± 37.9 mg/dl; P = 0.046) [73]. Other prebiotics, such as oligodextrans, lactose, resistant starches and their derivatives, lactoferrin-derived peptides, and N-acetyl chitooligosaccharides have also been identified as having the ability to maintain hypocholesterolemic effects in people with T2DM who are at high risk of developing CVD [74]. On the other hand, Giacco et al. reported that daily intake of 10.6 g of short-chain-fructooligosaccharides (sc-FOS) for 2 months by mild hypocholesterolemic individuals had no major effects on lipid metabolism compared with placebo groups (maltodextrin plus aspartame, 15 g/day) [75]. However, Kellow et al. emphasized that the results of these studies were limited because they evaluated relatively short-term prebiotic intervention periods and that large-scale trials of longer duration would be required to draw stronger conclusions [76].

### The anti-inflammatory effect

Gut permeability can be modified by an unbalanced intestinal microbiota, and bacteria and/or bacterial fragments, such as lipopolysaccharides (LPS), passing through the gut into the blood can lead to metabolic endotoxemia [77, 78]. LPS then binds to the cytokine receptors located in hepatocytes and adipocytes, thereby inducing pro-inflammatory cytokine release and insulin resistance. These molecules induce macrophage infiltration and result in the synthesis of inflammatory cytokines [77, 79], and induced cytokine signaling can then inhibit protein synthesis in order to enhance catabolism [79, 80]. Probiotics and prebiotics can enhance intestinal barrier functions, thus promoting the proliferation rate of beneficial or commensal gut microbes and impeding the progression of several gram-negative pathogens. In addition, probiotics and prebiotics can reduce LPS leakage and decrease pro-inflammatory cytokine production in adipose tissues. In total, we have acknowledged the need for future studies to evaluate whether this probiotic/synbiotic treatment can lead to a reduced pro-inflammatory state.

Moreover, gut microbiota composition can be mediated through probiotic supplementation by restoring glucose transporter-4 (GLUT4), PPAR-γ and lipogenic genes and pro-inflammatory marker (IL-6, TNF-α) expression in high-fructose fed rats. In mouse models, prebiotic supplementation can reduce low-grade inflammation caused by altered gut microbiota composition (decreased number of Firmicutes and increased abundance of Bacteroidetes) [40, 81]. Laminarin or fucoidan supplementation reduced Enterobacteriaceae population and abundance, which were clarified as attaching and effacing *E. coli* strains. Further studies confirmed that prebiotic supplementation could improve gut barrier functions in pigs. These prebiotics also markedly down-regulated the colonic mRNA expression of pro-inflammatory cytokines [82]. Increased expression levels of circulating GLP-1 and GLP-2 were observed in mouse models developed by diet control [44]. Prebiotic intake might also regulate enteroendocrine L-cell differentiation and the GLP-1 response [83, 84]. Increased GLP-2 production following a prebiotic diet was associated with an increased number of beneficial gut bacteria, improved integrity of the intestinal barrier, and lowered metabolic inflammation and endotoxemia. However, these effects have not been unraveled in humans [44, 83, 85–89]. Several studies have suggested that the gut microbiota can mediate the low-grade inflammation classically associated with metabolic disorders related to obesity by exerting an interesting “anti-inflammatory” effect [84, 90, 91].

Increased pro-inflammatory cytokine production was associated with aging [92], and the low-grade inflammation that is usually found in older people influenced the incidence of several age-associated diseases [22, 93]. Probiotics can modulate the immune system through phagocytosis, TH1 responses and pro-inflammatory cytokine production utilizing interleukin-10 (IL-10) [94]. Decreased IL-6 production can be detected in peripheral blood mononuclear cell (PBMC) in older people. In addition, a related prebiotic galacto-oligosaccharide mixture was shown to decrease Tumor Necrosis Factor-α (TNF-α), IL-1β and IL-6 and increase the production of IL-10 and natural killer cells [95]. An increase in serum CRP, which has been shown to correlate with circulating IL-6, can be evidence of systemic inflammation [96]. Several studies confirmed that the production of IL-6 was down-regulated and that the number of apoptotic T cells was increased in the lamina propria by the interaction of probiotics with inflamed intestinal tissues. A number of probiotic strains passing through the gastrointestinal tract can also induce anti-inflammatory cytokine production. The study advocated that this probiotic strain or its symbiotic combination can be beneficial by preventing lifestyle-associated, inflammation-associated, or gut microbiome-associated metabolic disorders through the amelioration of inflammatory status and gut microbial populations.

## Mechanisms of action

Currently, there is existing evidence from animal and human studies demonstrating the interaction between the modulation of the gut microbiota and various components of metabolic syndrome. Given that different strains and product formulations exist, the corresponding mechanisms are quite complex. In any event, the efficiency and mechanisms of probiotic effects are crucially determined by the link between probiotics and either the microbiota of the host or the immunocompetent cells of the intestinal mucosa [97]. To date, several assumptions about the mechanistic actions of probiotics and prebiotics that ameliorate MS, including modifications of gut microbial composition, involvements with energy homeostasis, the stimulation of insulin signaling, modulations of inflammatory signaling pathways, interference with the immune system, and the down-regulation of cholesterol levels, have been presented. Among the molecular mechanisms, this review focuses on short-chain fatty acids (SCFAs), bile-salt hydrolase (BSH), metabolic endotoxemia and the endocannabinoid (eCB) system, which are associated with the normalization of adipogenesis and the regulation of insulin secretion, fat accumulation, energy homeostasis, and plasma cholesterol levels, thus resulting in anti-obesity and anti-inflammatory effects, improving glycemic control and modulating serum lipids, as previously stated.

### SCFAs

It was reported that when probiotics settle in the gut, they ferment indigestible carbohydrates from food. Their action raises the level of SCFAs in the gut, principally acetate, propionate, and butyrate. Host recovery of SCFAs is generally efficient and occurs by both passive diffusion and mono-carboxylic acid transporters.

In the liver, butyrate can be metabolized into glutamate, glutamine and acetoacetate. Acetoacetate is a significant fuel resource for intestinal cells, and more than 70% of the oxygen consumption in isolated colonocytes was due to butyrate oxidation. As an intestinal nutrient, butyrate promotes the regeneration of intestinal cells to repair the intestinal mucosa, initiates the differentiation and apoptosis of normal intestinal cells, stimulates the synthesis of intestinal mucin glycoprotein, and strengthens the protective effect of the mucous layer. Acetate was reported to increase total cholesterol, and propionate increased glucose in the blood and reduced the hypercholesterolemia response caused by acetate. These effects were the consequence of propionate decreasing its involvement in cholesterol and fatty acid synthesis in the liver.

Recently, two orphan G Protein-Coupled Receptors (GPCRs), GPR41 (known as free fatty acid receptor3, FFAR3) and GPR43 (known as FFAR2), were found to be receptors for SCFAs, including acetate, propionate, and butyrate. FFAR2 was primarily activated by acetate and propionate, whereas FFAR3 was more often activated by propionate and butyrate [98]. FFAR2 and FFAR3 stimulated the release of intestinal hormone secretion, such as 5-hydroxytryptamine (5-HT), GLP-1 and PYY secretion. 5-HT is an important neurotransmitter involved in regulating gastrointestinal motility and secretory functions, which regulate intestinal permeability, promote intestinal peristalsis, and reduce the body’s absorption of food energy. GLP-1 had numerous physiological effects, including stimulation of glucose-dependent insulin secretion, augmentation of β-cell mass, and inhibition of glucagon release, gastric emptying, and food intake. Many investigations demonstrated that prebiotics increased GLP-1 release and improved metabolic inflammation and insulin resistance [87, 99, 100]. GLP-2 was co-secreted with GLP-1 and could enhance intestinal epithelial proliferation and reduce gut permeability [101]. In addition to the roles of GLP-2 in maintaining gut barrier integrity, slowing gastric emptying and intestinal motility, improving nutrient absorption, and enhancing immune function, GLP-2 in central neurons enhanced hepatic insulin sensitivity and played a key role in the control of glucose homeostasis [102]. PYY had several biological actions, including vasoconstriction, inhibition of gastric acid secretion, reduction of pancreatic and intestinal secretion, regulation of appetite and inhibition of gastric motility [103, 104].

Additional studies indicated that SCFAs were involved in the regulation of hepatic cholesterol synthesis [105, 106] and that the ingestion of SCFA-producing probiotics could increase SCFA influx into the liver, leading to the down-regulation of angiopoietin-like protein 4 (ANGPTL4). ANGPTL4 inhibited circulating lipoprotein lipase (LPL), which promoted lipid clearance [105, 107]. ANGPTL4 is also a downstream target gene of peroxisome proliferator activated receptors (PPARs), the agonists of which are widely utilized for the treatment of T2DM and CVD [108, 109]. PPAR-α mainly plays an important role in hepatic fatty acid oxidation, whereas PPAR-γ is the master regulator of adipogenesis [109].

### BSH

It was reported that an oral administration of probiotics significantly reduced cholesterol levels by as much as 22–33% and controlled elevated cholesterol levels in mice fed a fat-enriched diet. Enzymatic deconjugation of bile acids by BSH was proposed as an important molecular mechanism in cholesterol-lowering effects.

Bile salts can accelerate the decomposition of fat by increasing the acceptance area of lipase. In addition, bile salt molecules are concentrated in 3- to 6-micron diameter particles, called micro-particles (micelles), when they reach a certain concentration in the lumen. Decomposed lipid products, including fatty acids and cholesterol, are wrapped in internal micelles and form a soluble complex called mixed micelles. Therefore, bile salt acts a delivery vehicle, carrying insoluble decomposed lipid products to the surface of the intestinal mucosa, and these products are beneficial to the assimilation of digestive fat products in the intestine.

Deconjugated bile salts are less efficiently reabsorbed than conjugated bile salts, resulting in the excretion of larger amounts of free bile acids in feces. In addition, free bile salts are less efficient in the solubilization and absorption of lipids in the intestine. Therefore, deconjugation of bile salts could lead to a reduction in serum cholesterol either by increasing the demand for cholesterol during de novo synthesis of bile acids to replace those lost in feces or by reducing cholesterol solubility (and thereby cholesterol absorption through the intestinal lumen).

It was reported that BSH activity could hydrolyze conjugated glycodeoxycholic acid and taurodeoxycholic acid, leading to the deconjugation of glyco- and taurobile acids. Jones et al. evaluated the cholesterol-lowering effect of BSH utilizing *L. plantarum* 80 and *L. reuteri*, whereupon it was shown that the enzyme responsible for bile salt deconjugation in the enterohepatic circulation could be detected in probiotics indigenous to the gastrointestinal tract [110, 111]. Micelles, which play a role in the absorption of cholesterol in the intestine, are produced by bile salts, cholesterol, and phospholipids. By producing bile acids through the deconjugation of bile salts in the small intestine, probiotics prevent micelle production. When bile acid enters the enterohepatic circulation, probiotics hydrolyze the bile acid and bile salts through hydroxysteroid dehydrogenases, which are conjugated bile acid hydrolase enzymes. In doing so, the enterohepatic circulation of bile acids is disrupted.

### Metabolic endotoxemia

Lipopolysaccharides is a constituent of gram-negative bacterial cell walls and is the most potent inducer of inflammation. Recently, Cani and Delzenne demonstrated that excess dietary fat promoted an increase in plasma LPS concentrations, which they called “metabolic endotoxemia” because LPS plasma levels were much lower than those observed during septic shock [84]. Later, the relationship between metabolic endotoxemia and a high-fat diet was confirmed in a series of studies. Considering that LPS can affect inflammatory progress throughout the body and can interfere with both metabolism and the function of the immune system, it has been increasingly recognized that metabolic pathways and the innate immune system are functionally intertwined. In summary, these findings underline the role of fat intake and absorption in the growth of metabolic endotoxemia. Several independent experiments showed that gut bacteria are involved in the onset and progression of the inflammation related to MS by modulating plasma LPS levels. For instance, Cani et al. defined gut microbiota-derived LPS as a triggering factor in the early progress of inflammation and metabolic diseases, which was subsequently investigated in genetically and nutritionally obese mice by specifically modulating the composition of the gut microbiota. Altogether, they indicated that specific microbe-associated molecular patterns (MAMPs), such as LPS, play a critical role in the onset the diseases related to MS [40, 49, 83–85, 100, 112].

The host symbiotic bacteria affect the immune system through the interaction between their pathogen-associated microbial patterns (PAMPs) (including LPS, lipoteichoic acids (LTK) of cell walls of bacteria, flagellin and single- or double-stranded RNA and DNA) and specific toll-like receptors (TLRs) of epithelial and dendritic cells (DCs) of the digestive tract [94, 113, 114]. TLRs are a member of the integral membrane pattern-recognition receptor family, which plays a major role in innate immunity by integrating signals from microbiota-host interactions; these interactions are vital to maintaining this balance [115]. In summary, the innate immune system detects LPS via its interaction with the CD14/TLR4 complex at the surface of innate immune cells.

A series of studies indicated that modulation of the gut microbiota (e.g., by probiotics) reduced metabolic endotoxemia and the cecal content of LPS; improved low-grade inflammation, steatosis, glucose intolerance and insulin sensitivity; decreased body weight gain; and prevented the development of MS both in obese animal models and in clinical studies [85, 116–118]. Moreover, prebiotic-induced changes in the gut microbiota could also abolish obesity-related metabolic features. Dietary intervention with oligofructoses could regulate the composition of the gut microbiota in a complex way in response to a high-fat diet or genetic obesity.

Some recent studies underlined key mammalian host–gut microbial relationships, inferring that the gut microbiota played a vital role in metabolic pathways [119]. Cani et al. suggested that plasma LPS levels interacted negatively with *Bifidobacterium* spp. among the analyzed gut bacteria. In other words, *Bifidobacterium* spp. supplementation was shown to improve mucosal barrier function and decrease intestinal endotoxin levels [120–122]. Furthermore, mice fed prebiotic dietary fiber (FOS) had normal endotoxemia. The precise mechanism by which FOS reduces metabolic endotoxemia and systemic inflammatory levels remains elusive. However, *Bifidobacterium* spp. is recognized as the main gut microbiota linked to the positive effects of FOS supplementation. In fact, *Bifidobacterium* spp. supplementation has been linked to lowering bacterial translocation and endotoxemia, leading to decreased inflammatory cascade activation [120–122]. Thus, specific strategies for modifying the gut microbiota by probiotics and prebiotics could be potential targets for reducing the impact of high-fat feeding on the occurrence of metabolic diseases.

### eCB system

Another potential mechanism involved in the modulation of the gut microbiota on the development of MS and other related disorders is the endocannabinoid system (eCB), which is a summation of several bioactive lipids, enzymes and distinct types of receptors [123]. Two kinds of lipids are the most studied, including N-arachidonoylethanolamide (anandamide; AEA) and 2-arachidonoylglycerol (2-AG) [124]. Subsequently, there are two primary enzymes, monoacylglycerol lipase (MAGL) and fatty acid amide hydrolase (FAAH), that regulate the metabolism of AEA and 2-AG, respectively, from cell membrane phospholipids after cell stimulation [83]. After their release, eCBs interact with Gi-coupled receptors, cannabinoid receptor 1 (CB1) and Go-coupled receptors CB2, which are also targeted by the principal active component of *Cannabis sativa*, ∆9-tetrahydrocannabinol [125].

Several studies have shown that eCB system impacts the regulation of energy homeostasis and the control of lipid and glucose metabolism at several levels [126, 127]. Obesity is characterized by greater eCB system responsiveness. In other words, hyperactivity of eCB is associated with altered expression of CB1 mRNA, decreased levels of enzymes, and increased plasma eCB, adipose tissue eCB levels, and CB1 activity. This hyperresponsiveness brings about unbalanced energy intake, which is conducive to excessive intra-abdominal fat accumulation and is related to the development of MS [128].

The gut microbiota can determine gut permeability and adipose tissue physiology through LPS-eCB system regulatory loops. In other words, the massive expansion of adipose tissues upon obesity is characterized by low-grade inflammation, which is possibly controlled by the gut microbiota (via LPS), and LPS can stimulate eCB synthesis. The eCB system is hyper-activated in the intestine, which leads to increases in gut permeability, plasma LPS levels and systemic inflammation. The interaction between the eCB system and the gut microbiota modulates adipogenesis directly by acting upon adipose tissue and indirectly by increasing plasma LPS levels.

Regulating the gut microbiota with prebiotics in ob/ob mice decreases CB1 mRNA expression in adipose tissue, reduces LPS levels in plasma and increases adipocyte lipogenesis and differentiation; these effects indicate that the gut microbiota may have critical functions in adipose tissue plasticity during obesity and may determine adipose tissue physiology through LPS-eCB system regulatory loops [89]. Recently, Cani et al. found that specific modulation of the gut microbiota with prebiotics promotes normalization of eCB system responsiveness in both the gut and in adipose tissue. These effects are deeply related to decreases in gut permeability, metabolic endotoxemia and fat mass development. Therefore, prebiotic-induced changes in the gut microbiota reduce both adipose tissue and intestinal eCB system responsiveness, consequently normalizing adipogenesis and improving the gut barrier.

Modulation of the intestinal microbiota with specific probiotics has been shown to upregulate CB2 receptor expression in rodents. Bermudez-Silva et al. [129] indicated that CB2 receptor activation ameliorates glucose tolerance in rats. CB2 receptor expression is positively regulated with intestinal counts of Lactobacillus supplement and negatively regulated with counts of Clostridium supplement. Later, Rousseaux et al. [130] demonstrated that administration of *L. acidophilus* increases CB2 receptor expression in mouse colon. However, it should be noted that even if potent interrelation exists between the composition of the gut microbiota and elements controlling the eCB system, the direct involvement of specific gut microbes and/or of microbial metabolites needs further elucidation.

### Summary

The gut microbiota is now considered to be involved in the regulation of multitudinous physiological pathways and to impact different host functions [131]. Given the developing knowledge of how probiotics and prebiotics interact with the gut microbiota, there has been a growing interest in exploring the effect of probiotics and prebiotics on specific constituents of MS. Four main mechanisms have been proposed in this review to explain the action of probiotics. The first mechanism is raising bacteria-derived SCFAs, which activates GPR-43 on L cells and triggers the secretion of GLP-1 and GLP-2. These hormones exhibit an enormous variety of metabolic and proliferative actions. The second mechanism is increasing BSH activity. Bacterial BSH enzymes in the gut influence host physiological processes, resulting in decreases in body weight gain and plasma cholesterol levels. The third mechanism is leveraging the anti-inflammatory function of probiotics, which improves low-grade inflammation, steatosis, glucose intolerance and insulin sensitivity. The fourth mechanism is down-regulation of eCB system responsiveness, which impacts the regulation of energy homeostasis and the normalization of adipogenesis.

## Conclusion

Accumulating evidence suggests that gut microbiota plays a significant role in the initiation and progression of MS. The gut microbiota was proven to modulate plasma glucose, appetite, serum lipids and pro-inflammation. In addition, prebiotics or probiotics, which are widely used to manipulate the microbiota, can reduce low-grade intestinal inflammation and improve gut barrier integrity to reduce plasma glucose and serum lipid levels, induce weight loss and decrease insulin resistance. Based on these current achievements, the gut microbiota may be a potential therapeutic target for MS. However, clinical trials addressing the efficacy and efficiency of current or potential treatments on therapeutic applications in metabolic syndrome are needed.

## Abbreviations

MS: metabolic syndrome
SCFAs: short-chain fatty acids
BSH: bile salt hydrolase
eCB: endocannabinoid
GI: gastrointestinal
OS: oligosaccharides
NDO: non-digestible carbohydrates
ITF: inulin-type fructans
GOS: galacto-oligosaccharides
FOS: fructooligosaccharides
XOS: xylo-oligosaccharides
POS: pectic oligosaccharides
LG: *Lactobacillus* *gasseri*
HFD: high-fat diet
PPAR: peroxisome proliferator activated receptor
ITF: inulin-type fructans
PYY: peptide YY
GLP: glucagon-like peptide
BMI: body mass index
FM: fermented milk
T2DM: type 2 diabetes mellitus
G6pc: glucose-6-phosphatase
hs-CRP: high-sensitivity C-reactive protein
FPG: fasting plasma glucose
GSH: glutathione, r-glutamyl cysteinyl +glycine
CVD: cardiovascular disease
HFCD: high-fat high-cholesterol diet
CU: curvatus
PL: plantarum
sc-FOS: short-chain-fructooligosaccharides
LPS: lipopolysaccharides
GLUT: glucose transporter
IL: interleukin
PBMC: peripheral blood mononuclear cell
TNF: tumor necrosis factor
GPCR: G protein-coupled receptor
FFAR: free fatty acid receptor
5-HT: 5-hydroxytryptamine
ANGPTL: angiopoietin-like protein
LPL: lipoprotein lipase
PPARs: peroxisome proliferator activated receptors
MAMPs: microbes-associated molecular patterns
PAMPs: pathogen-associated microbial patterns
LTK: lipoteichoic acids
TLRs: toll-like receptors
DCs: dendritic cells
AEA: arachidonoylethanolamide
2-AG: 2-arachidonoylglycerol
MAGL: monoacylglycerol lipase
FAAH: fatty acid amide hydrolase
CB1: cannabinoid receptor 1
